# Skull Base Collision Tumors: Giant Non-functioning Pituitary Adenoma and Olfactory Groove Meningioma

**DOI:** 10.7759/cureus.44710

**Published:** 2023-09-05

**Authors:** Mehmet V Aydin, Kivanc Yangi, Ezgi Toptas, Seckin Aydin

**Affiliations:** 1 Neurological Surgery, Prof. Dr. Cemil Taşcıoğlu City Hospital, Istanbul, TUR; 2 Pathology, Prof. Dr. Cemil Taşcıoğlu City Hospital, Istanbul, TUR

**Keywords:** sella turcica, olfactory groove meningoma, nonfunctioning pituitary adenoma, skull base collision tumors, collision tumor

## Abstract

A collision tumor complex is composed of at least two different tumors, benign or malignant, with at least two different histopathological features located adjacent to each other in the exact anatomical localization. Pathologies such as meningiomas, pituitary adenomas, gliomas, and schwannomas may be involved in a collision tumor complex. However, co-occurrence of pituitary adenomas and meningiomas as skull base collision tumors is rare. Here, we present a 65-year-old male patient who presented with olfactory groove meningioma and non-functioning pituitary adenoma as a collision tumor. The patient was admitted with a headache and right-sided vision loss. The patient's first neurologic examination was consistent with temporal anopsia in the right eye. Subsequent contrast-enhanced cranial MRI revealed a 65x55x40 mm heterogeneously contrast-enhanced lesion in the anterior skull base extending from the sellar region to the corpus callosum. Because of the tumor size, a two-staged operation was planned. First, the tumor was partially excised via a right frontal craniotomy with a transcranial approach, and the tumor in the sellar region was left as a residue. The pathology reports after the first surgery showed pituitary adenoma and meningeal epithelial type meningioma (WHO Grade I). The residual tumor tissue was resected seven months later via an endoscopic endonasal approach, except for the part that invaded the right anterior cerebral artery. The optic nerve was decompressed. The patient was then referred to the radiation oncology clinic for radiosurgery. Collision tumors should be considered in the differential diagnosis in preoperative evaluation and surgical planning when heterogeneously contrast-enhanced areas significantly localized adjacent to each other are seen on cranial MRI. On the other hand, when the surgeon encounters sudden changes in the appearance or consistency of the tumor during the surgery, they should suspect these tumor complexes. The diagnosis of collision tumors is quite challenging but is of great importance regarding the patient's need for postoperative radiation therapy or the recurrence characteristics of tumors. However, more studies are needed on these complexes' etiology, surgical planning, and postoperative management.

## Introduction

A collision tumor complex comprises at least two distinct tumors with different histological features located adjacent to each other [1]. One metastasis and one new primary neoplasm, two different metastases, or two new primary neoplasms may be seen as a collision tumor complex [2]. Skull base collision tumors are extremely rare pathologies. Meningiomas, pituitary adenomas, gliomas, and schwannomas can be presented as collision tumors in this region. Meningiomas and gliomas have been reported as the most common neuropathologies associated with collision tumor complexes [3].

The co-occurrence of meningiomas and pituitary adenomas as a collision tumor complex is an infrequent condition. We present a case of a sella turcica collision tumor complex in a 65-year-old male patient who is otherwise healthy.

## Case presentation

A 65-year-old male patient presented with headache and right homonymous hemianopsia, which started a month previously. The patient had no history of chronic diseases, never smoked, or did not use illicit drugs. The neurologic examination was unremarkable except for the visual disturbances. Right homonymous hemianopsia was verified in the visual field evaluation.

**Figure 1:**
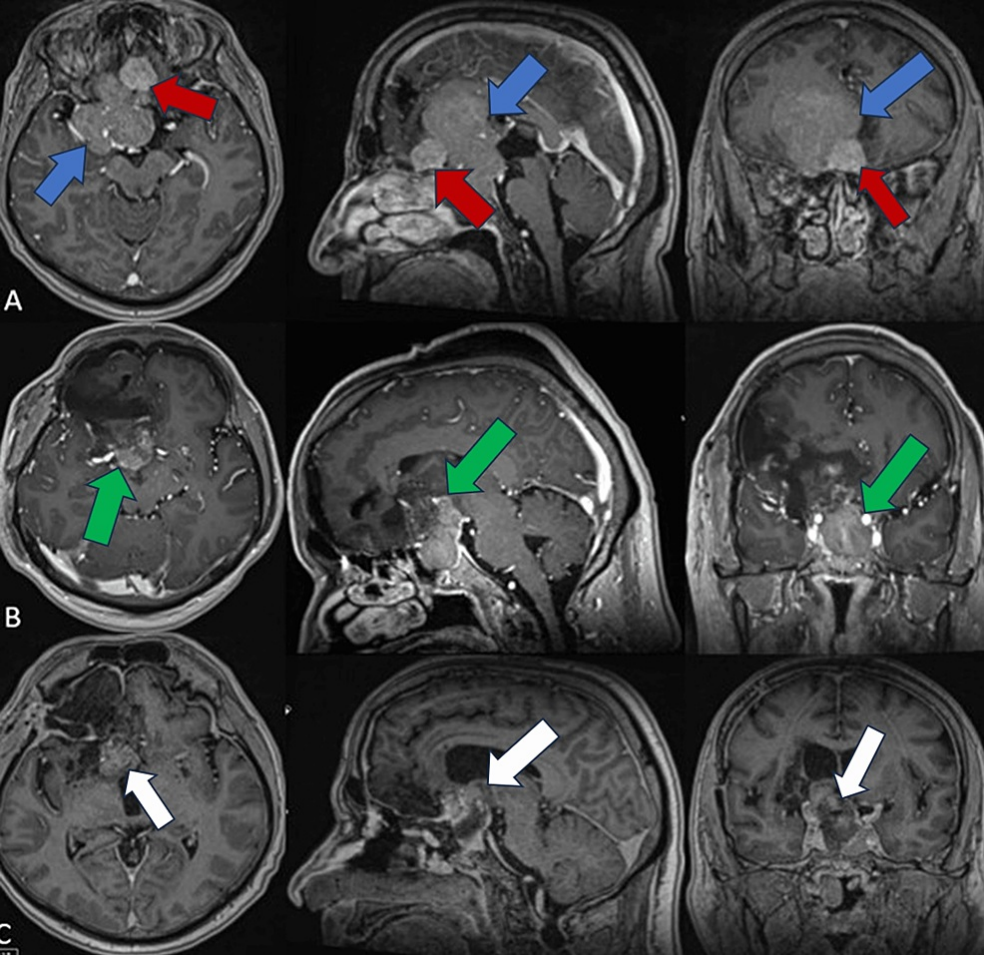
Preoperative, first, and second postoperative contrast-enhanced T1-weighted cranial MRI of a patient with non-functioning pituitary adenoma and co-existing olfactory groove meningioma. A: Preoperative contrast-enhanced T1-weighted cranial MRI of a patient with collision tumor; axial, sagittal and coronal scans, respectively. A heterogeneously contrast-enhanced mass lesion of approximately 65x55x40 mm in size with lobulated contours, filling the sellar lobe and extending superiorly to the convexity level of the frontal lobe, was observed (blue arrows). A well-circumscribed nodular lesion is observed in the anterior part of the frontal lobe with a heterogeneous appearance on the postcontrast series at a distance of approximately 2 cm from the other mass lesion at the level of the falx cerebri (red arrows). The lesion was evaluated as first plane olfactory groove meningioma. B: First postoperative contrast-enhanced T1-weighted cranial MRI; axial, sagittal, and coronal scans, respectively. The sellar lesion, observed on the preoperative MRI scans, was largely resected. Postoperative changes were seen in this imaging. Within the sella, a 37x30 mm residual cystic necrotic lesion was observed (green arrows), filling the sella and displacing both middle cerebral artery (MCA) M1 parts superiorly. C: Second postoperative contrast-enhanced T1-weighted cranial MRI; axial, sagittal, and coronal scans, respectively. It was observed that the lesion was mostly excised. A residual lesion with a diameter of approximately 17 mm was observed (white arrows). It was seen that the sellar and suprasellar components of the tumor were removed, and the optic nerve was decompressed, but there was a residue in the anterior cerebral artery (ACA) A1 segment.

A heterogenously contrast-enhanced space-occupying lesion, 65x55x40 mm in diameter, was observed on the contrast-enhanced T1-weighted cranial MRI. The lesion was arising from the tuberculum sella, occupying the anterior skull base, invading the cavernous sinuses bilaterally, and extending towards the right frontal cortex (Figure 1A). The lesion was in a close relationship with the right anterior cerebral artery A1-segment. On the other hand, the lesion was placed proximal to the right middle cerebral artery M1 segment.

Adrenocorticotropic Hormone (ACTH), cortisol, LH, Free T3, Free T4, and TSH values ​​were below the threshold in the anterior pituitary hormone panel. On the contrary, the prolactin level was above the threshold. ACTH suppression test was performed and resulted in a low cortisol value. As a result, 40 mg/day methylprednisolone and 50 mg/day L-thyroxine were started by Endocrinology due to low T4 and cortisol levels.

Two-staged surgical treatment was planned. First, the tumor was exposed via a right frontotemporal craniotomy. In the beginning, tissue samples were gathered from the soft component of the tumor. Intraoperative frozen-section results were consistent with pituitary adenoma. The tumor was carefully dissected from the middle cerebral artery branches, and tuberculum sella was exposed. The part of the lesion that adhered to the anterior cerebral artery (ACA) A2 branches was left as a residue. The optic chiasm was exposed. When the olfactory groove was exposed, the lesion's consistency was changed. A new tissue sampling from the harder part of the tumor was gathered and sent to the intraoperative frozen section. The result was compatible with meningioma. The rigid part of the lesion was gross-totally excised.

Postoperative cranial MRI revealed that the parts of the tumor extending to the frontal lobe and displacing the corpus callosum, were resected, and residue remained in the sellar and suprasellar regions (Figure 1B). In the patient's first postoperative visual field evaluation, improvement was detected in the right homonymous hemianopsia when compared to the preoperative evaluation. The postoperative neurological exam and hormone panel were unremarkable. The patient was discharged on the ninth postoperative day without complications until the second surgery was planned.

**Figure 2:**
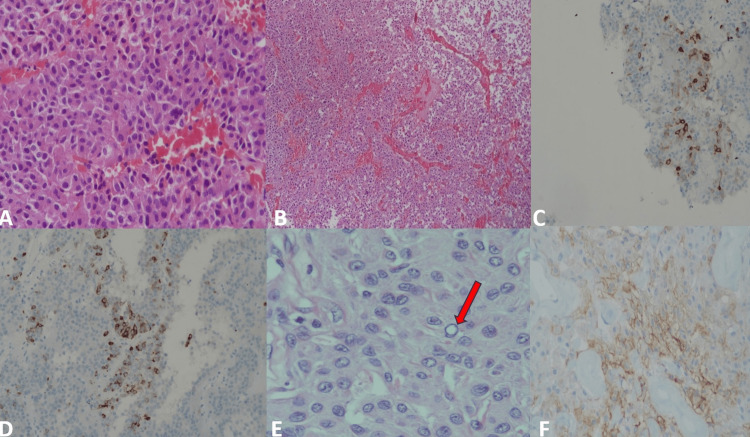
Pathologic specimen pictures of the biopsy material from the first operation A: Neoplastic cells with eosinophilic cytoplasm, round/oval nuclei with indistinct nucleoli (H&E x 200); B: Tumoral proliferation consists of uniform cells with a distinctive diffuse pattern and vascular framework (H&E x200); C: Focal positivity with luteinizing hormone (LH), LH x200; D: Focal positivity with follicle-stimulating hormone (FSH), FSH x200; E: Neoplastic cells with pseudo-inclusions (red arrow), H&E x200; F: Epithelial membrane antigen (EMA) positivity, EMA x400. H&E: hematoxylin & eosin

Three different biopsy materials were gathered during the first operation. The histopathological examination of the first two samples revealed that the tumor had a prominent vascular framework and consisted of cells with round/oval nuclei and extensive eosinophilic, granular cytoplasm, focal/slight pleomorphism, and indistinct nucleoli (Figures 2A-2B). In the immunohistochemical study, the tumor cells showed positive staining with synaptophysin, GATA binding protein 3 (GATA3), steroidogenic factor-1 (SF1), and dot-like staining with pan-cytokeratin (CKPan) and focal positivity with luteinizing hormone (LH) (Figure 2C) and follicle-stimulating hormone (FSH) (Figure 2D). The tumor cells showed negative results with prolactin, ACTH, and growth hormone (GH). Ki-67 proliferation index was low (2%). Based on these findings, the biopsy was reported as a "pituitary neuroendocrine tumor/pituitary adenoma-gonadotropic type.”

The histopathological examination of the third biopsy material showed uniform tumoral infiltration with indistinct cytoplasmic borders and syncytial growth pattern. Tumor tissue consisted of cells with eosinophilic cytoplasm, indistinguishable nucleoli which contained pseudo-inclusions (Figure 2E), and round nuclei. Necrosis and surrounding parenchymal invasion were not observed. In the immunohistochemical study, positive staining with epithelial membrane antigen (EMA) (Figure 2F) and focal mild/moderate staining with progesterone were observed in tumor cells. The Ki67 proliferation index was found to be 20%. No staining was observed with glial fibrillary acidic protein (GFAP). Based on these findings, the biopsy was reported as “meningothelial menegioma (WHO grade I),” the most common meningioma variant.

Seven months after the first operation, the patient was re-operated via an endoscopic endonasal transsphenoidal approach to remove the residual lesion. The lesion destroyed the sellar base. After opening the sellar dura, tumor resection was started with the help of an endoscopic curette and aspirator. The tumor's consistency was moderately firm. There was no obvious cavernous sinus invasion. The optic chiasm was decompressed. Most of the suprasellar component of the tumor was excised. The tumor was in a close proximity with the right ACA A1 segment. This part of the tumor was left as a residue to prevent vascular damage.

Postoperative contrast-enhanced T1-weighted cranial MRI revealed that the sellar and suprasellar components of the tumor were removed. The optic nerve was decompressed. However, residue tumoral tissue was observed near the ACA A1 segment (Figure 1C)

In the second postoperative visual field evaluation, significant improvement was observed in the right homonymous hemianopsia compared to preoperative and first postoperative evaluations. The patient's postoperative neurological examination and hormone panel were unremarkable. No endocrine abnormalities were observed postoperatively. The patient's medications were continued in the postoperative period as recommended by endocrinology. The patient was referred to radiation oncology for radiosurgery. No increase in residual lesion size was observed in the patient's postoperative third and sixth-month controls.

**Figure 3:**
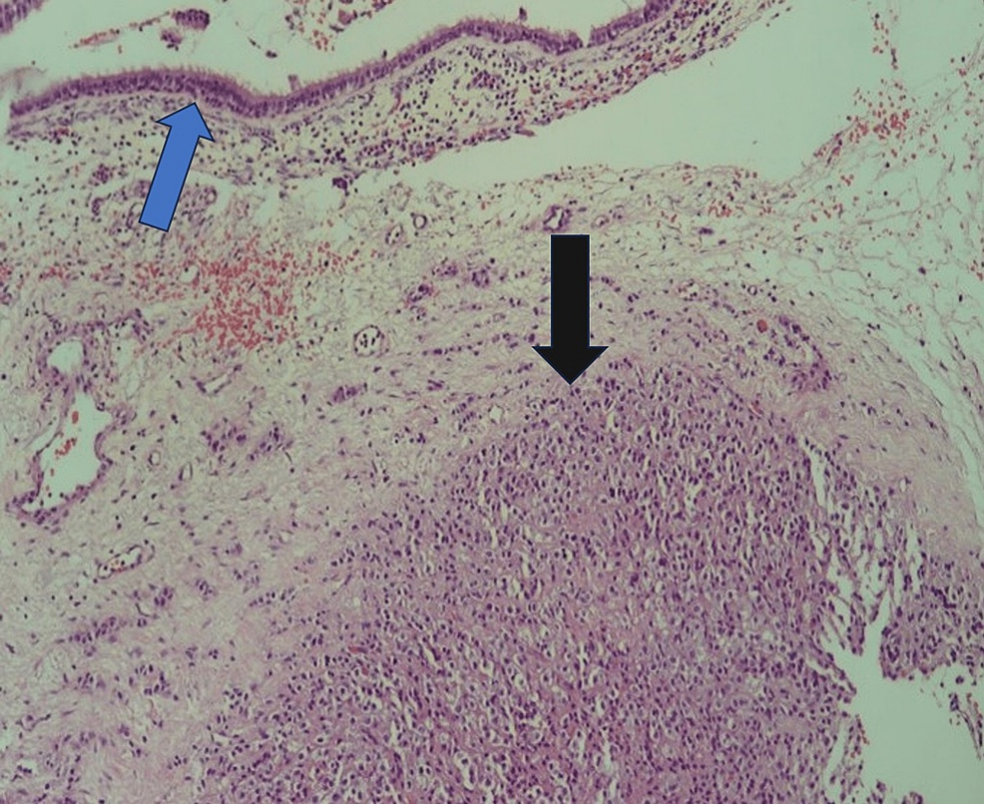
Pathologic regimen from the second operation, hematoxylin & eosin, x100 Microscopic picture of the biopsy material from the second operation. Note the association between the sinus mucosa cells (blue arrow) and the pituitary adenoma neoplastic cells (black arrow)

The histopathological examination of the biopsy materials of the second operation showed sinus mucosa containing respiratory-type epithelium on the surface, and the tumoral proliferation was compatible with pituitary adenoma in the subepithelial area (Figure 3). Tumor cells had eosinophilic, granular cytoplasm, indistinct nucleoli, and centrally located round/oval nuclei. Neoplastic cells were also observed between bone trabeculae, and the tumor seemed to invade the bony tissue. In the immunohistochemical study, neoplastic cells exhibited the same staining pattern as the pituitary adenoma identified after the first operation. Based on these findings, the biopsy was reported as a “pituitary neuroendocrine tumor/pituitary adenoma-gonadotropic" type.

## Discussion

Collision brain tumor complexes of two primary brain tumors are extremely rare entities. This situation can be seen in various genetic syndromes or may occur after cranial radiation therapy. Collision tumor complexes, seen without a history of cranial radiation therapy or genetic syndromes, are uncommon [4]. According to the literature, the formation of a second neoplasm may be induced by tissue alterations created by a preceding neoplasm. Furthermore, it has also been hypothesized that two distinct tumors may coincidentally occur without any certain pathophysiological explanation [5,6]. Meningiomas and gliomas are the most common components of an intracranial collision tumor complex. In this case, we present a patient with two distinct primary brain tumors that occurred without a history of cranial radiation therapy or a known genetic syndrome.

The relationship between pituitary adenomas and co-occurring meningiomas is unclear. Pituitary adenomas can be seen together with meningiomas after cranial radiotherapy, as stated in the literature. A study by Minniti et al. reported that the risk of meningioma occurrence in the radiated intracranial field is increased by 2.4%, especially in the following 20 years [5]. On the other hand, two other studies reported that these tumors may occur independently and simultaneously without a preceding cranial radiotherapy history [6,7]. There is no consensus on which tumors appear first, whether they transform into each other or require different treatment modalities. Another study showed that prolactin secretion is essential for the development of secondary meningiomas in cases of pituitary adenoma [8]. Although we do not know which tumor occurred first in our case, this study may be a guide for further studies.

Furthermore, GH is the most commonly seen stimulant hormone for meningioma development [9]. GH and somatostatin may act as a stimulant on the dura and arachnoid cells and cause meningioma formation [10]. As in our case, the co-existence of a non-secreting pituitary adenoma and an olfactory groove meningioma is a much rarer situation. On the other hand, meningiomas are the most common tumors associated with multiple intracranial tumors. This may be related to meningiomas being common pathologies that do not require an extended clinical evaluation before diagnosis.

The co-existence of these two tumors is unusual and challenging to manage. After a lengthy discussion, our neurosurgical team evaluated this tumor as an olfactory groove meningioma because of its irregular borders and radiological appearance. However, the hormone panel indicated a non-functioning pituitary adenoma. It is known that sellar and suprasellar meningiomas can mimic pituitary adenomas radiologically. During the first surgery, we came across a sudden change in the consistency of the tumor and we collected different biopsy materials from two different parts of the tumor. In this case, where the pathology report resulted in meningeal epithelial type meningioma and pituitary adenoma, the importance of collecting and sending biopsy samples from different points becomes evident. A different pathology report could have been encountered if tissue samples from only one part of the tumor had been sent. Accurate pathological results are essential with regard to post-operative patient follow-up, possible radiotherapy, tumor recurrence, and radiotherapy dosage adjustment.

In most articles previously reported in the literature, different treatment approaches were applied to these tumor complexes, and the two tumors' surgical treatments were planned independently. As we preferred in our case, pituitary adenomas can be managed through the endoscopic endonasal transsphenoidal approach, and the transcranial approach may be the treatment of choice for olfactory groove meningioma. However, in some cases, as long as tumor size and location allow, performing these cases with a single-session endoscopic endonasal transsphenoidal or single-session transcranial approach may be considered. Determining the appropriate surgical approach is crucial to avoid severe complications.

## Conclusions

Sella turcica collision tumors are extremely rare pathologies. The accurate diagnosis may not be made preoperatively, which may mislead the surgeon. This poses a risk for the postoperative follow-up of the patient. Collision tumors should be considered in the differential diagnosis when heterogeneously contrast-enhanced lesions are detected on contrast-enhanced T1-weighted cranial MRI. For accurate treatment planning, biopsy samples from different localizations of the tumors should be sent intraoperatively; especially if sudden differences in the size and consistency of the tumor are suspected, the preliminary diagnosis must be confirmed by pathological diagnosis. This is essential in determining the disease's prognosis, estimating the probability of tumor recurrence, and the patient's suitability for chemoradiotherapy. Considering these two different tumors, surgical planning is of great importance. It is highly controversial whether two tumors can be excised in a single surgical session and which tumor should be excised first. We need more experience and studies to understand these tumor complexes' molecular and genetic backgrounds.
